# Supplementary material for: Increasing tendency of urine protein is a risk factor for rapid eGFR decline in patients with CKD: A machine learning-based prediction model by using a big database
Source: PLoS One. 2020 Sep 17;15(9):e0239262. doi: 10.1371/journal.pone.0239262 (PMC7497987; doi:10.1371/journal.pone.0239262)
Supplement: S1 Table — (DOCX) [file pone.0239262.s001.docx]

**Supplementary Table 1. Average blood pressure and laboratory data for 90, 180, and 360 days prior to the baseline**

| Variables | All  n, 19,732 | RD group  n, 9,866 | Non-RD group  n, 9,866 | p value |
| --- | --- | --- | --- | --- |
| 90 days prior to the baseline | | | | |
| SBP (mmHg) | 132, 23 | 136, 24 | 129, 22 | < 0.001 |
| DBP (mmHg) | 73, 14 | 74, 14 | 72, 13 | < 0.001 |
| eGFR (ml/min/1.73m^2^) | 39.9, 24.6 | 39.5, 24.7 | 40.3, 24.5 | 0.021 |
| Serum creatinine (mg/dL) | 2.26, 2.15 | 2.24, 2.03 | 2.29, 2.27 | 0.005 |
| BUN (mg/dL) | 28.9, 15.8 | 29.6, 16.1 | 28.2, 15.4 | < 0.001 |
| Hemoglobin (mg/dL) | 11.5, 2.1 | 11.5, 2.0 | 11.6, 2.2 | < 0.001 |
| Hematocrit (%) | 35.0, 6.1 | 34.8, 5.8 | 35.2, 6.3 | < 0.001 |
| Serum T-C (mg/dL) | 182, 46 | 186, 48 | 177, 44 | < 0.001 |
| Serum TG (mg/dL) | 142, 88 | 151, 97 | 134, 77 | < 0.001 |
| Serum uric acid (mg/dL) | 6.2, 1.8 | 6.4, 1.8 | 6.0, 1.7 | < 0.001 |
| Urine protein * | 1.9, 1.8 | 2.3, 1.9 | 1.4, 1.6 | < 0.001 |
| Urine protein ** | 1 [0, 3] | 2 [0, 4] | 1 [0, 2.5] |  |
| 180 days prior to the baseline | | | | |
| SBP (mmHg) | 132, 23 | 135, 23 | 129, 22 | < 0.001 |
| DBP (mmHg) | 73, 13 | 74, 13 | 72, 13 | < 0.001 |
| eGFR (ml/min/1.73m^2^) | 39.7, 24.0 | 39.2, 23.7 | 40.3, 24.2 | 0.003 |
| Serum creatinine (mg/dL) | 2.27, 2.2 | 2.22, 2.02 | 2.31, 2.31 | 0.001 |
| BUN (mg/dL) | 28.7, 15.1 | 29.4, 15.2 | 28.0, 14.9 | < 0.001 |
| Hemoglobin (mg/dL) | 11.5, 2.1 | 11.5, 2.0 | 11.6, 2.1 | < 0.001 |
| Hematocrit (%) | 35.1, 5.9 | 34.9, 5.7 | 35.2, 6.1 | < 0.001 |
| Serum T-C (mg/dL) | 182, 45 | 187, 47 | 178, 43 | < 0.001 |
| Serum TG (mg/dL) | 144, 86 | 152, 95 | 135, 75 | < 0.001 |
| Serum uric acid (mg/dL) | 6.2, 1.7 | 6.4, 1.7 | 6.0, 1.7 | < 0.001 |
| Urine protein * | 1.9, 1.8 | 2.3, 1.8 | 1.4, 1.5 | < 0.001 |
| Urine protein ** | 1.5 [0, 3] | 2.25 [0.3, 4] | 1 [0, 2.5] |  |
| 360 days prior to the baseline | | | | |
| SBP (mmHg) | 132, 22 | 135, 22 | 129, 21 | < 0.001 |
| DBP (mmHg) | 73, 13 | 74, 13 | 72, 12 | < 0.001 |
| eGFR (ml/min/1.73m^2^) | 40.2, 23.6 | 39.8, 23.1 | 40.6, 24.1 | 0.038 |
| Serum creatinine (mg/dL) | 2.24, 2.17 | 2.17, 2.00 | 2.31, 2.32 | 0.006 |
| BUN (mg/dL) | 28.3, 14.5 | 28.8, 14.3 | 27.7, 14.6 | < 0.001 |
| Hemoglobin (mg/dL) | 11.6, 2.0 | 11.5, 2.0 | 11.6, 2.1 | 0.005 |
| Hematocrit (%) | 35.1, 5.7 | 35.0, 5.6 | 35.3, 5.9 | < 0.001 |
| Serum T-C (mg/dL) | 183, 44 | 187, 45 | 179, 42 | < 0.001 |
| Serum TG (mg/dL) | 145, 84 | 153, 92 | 136, 73 | < 0.001 |
| Serum uric acid (mg/dL) | 6.2, 1.6 | 6.4, 1.6 | 6.1, 1.6 | < 0.001 |
| Urine protein * | 1.8, 1.7 | 2.3, 1.8 | 1.4, 1.5 | < 0.001 |
| Urine protein ** | 1.5 [0, 3] | 2.1 [0.4, 4] | 1 [0, 2.4] |  |

Mean, standard deviation, Value, %

* Continuous value of urine protein test by dipstick

** Semi-quantity test of urine protein test by dipstick 50% [25%, 75%],

0; -, 1; +, 2; +, 3; ++, 4; +++, 5; ++++

RD; rapid decline, SBP; systolic blood pressure, DBP; diastolic blood pressure, eGFR; estimated glomerular filtration rate, BUN; blood urea nitrogen, T-C; total cholesterol, TG; triglyceride
