# Supplementary material for: Increasing tendency of urine protein is a risk factor for rapid eGFR decline in patients with CKD: A machine learning-based prediction model by using a big database
Source: PLoS One. 2020 Sep 17;15(9):e0239262. doi: 10.1371/journal.pone.0239262 (PMC7497987; doi:10.1371/journal.pone.0239262)
Supplement: S2 Table — (DOCX) [file pone.0239262.s002.docx]

**S2 Table. Exponentially smoothed average of blood pressure and laboratory data for 7 and 30days**

| Variables | All  n, 19,732 | RD group  n, 9,866 | Non-RD group  n, 9,866 | p value |
| --- | --- | --- | --- | --- |
| 7 days | | | | |
| SBP (mmHg) | 132, 22 | 136, 23 | 129, 22 | < 0.001 |
| DBP (mmHg) | 73, 13 | 74, 13 | 72, 13 | < 0.001 |
| eGFR (ml/min/1.73m^2^) | 39.9, 24.3 | 39.4, 24.2 | 40.3, 24.4 | 0.017 |
| Serum creatinine (mg/dL) | 2.27, 2.14 | 2.24, 2.02 | 2.29, 2.26 | 0.002 |
| BUN (mg/dL) | 28.9, 15.4 | 29.6, 15.6 | 28.2, 15.2 | < 0.001 |
| Hemoglobin (mg/dL) | 11.5, 2.1 | 11.5, 2.0 | 11.6, 2.1 | < 0.001 |
| Hematocrit (%) | 34.9, 6.0 | 34.8, 5.7 | 35.1, 6.2 | < 0.001 |
| Serum T-C (mg/dL) | 182, 45 | 186, 47 | 177, 43 | < 0.001 |
| Serum TG (mg/dL) | 143, 86 | 152, 95 | 134, 75 | < 0.001 |
| Serum uric acid (mg/dL) | 6.2, 1.7 | 6.4, 1.7 | 6.0, 1.7 | < 0.001 |
| Urine protein * | 1.9, 1.8 | 2.3, 1.8 | 1.4, 1.5 | < 0.001 |
| Urine protein ** | 1.6 [0.0, 3.0] | 2.3 [0.3, 4.0] | 0.9 [0.0, 2.5] |  |
| 30 days | | | | |
| SBP (mmHg) | 132, 20 | 135, 21 | 129, 20 | < 0.001 |
| DBP (mmHg) | 73, 12 | 74, 12 | 72, 12 | < 0.001 |
| eGFR (ml/min/1.73m^2^) | 40.6, 23.6 | 40.4, 23.1 | 40.8, 24.1 | 0.517 |
| Serum creatinine (mg/dL) | 2.23, 2.16 | 2.15, 1.97 | 2.31, 2.32 | 0.062 |
| BUN (mg/dL) | 28.2, 14.3 | 28.7, 14.0 | 27.8, 14.5 | < 0.001 |
| Hemoglobin (mg/dL) | 11.5, 2.0 | 11.5, 1.9 | 11.6, 2.0 | 0.016 |
| Hematocrit (%) | 35.0, 5.6 | 34.9, 5.4 | 35.1, 5.8 | < 0.001 |
| Serum T-C (mg/dL) | 183, 42 | 187, 44 | 179, 40 | < 0.001 |
| Serum TG (mg/dL) | 145, 81 | 153, 89 | 136, 71 | < 0.001 |
| Serum uric acid (mg/dL) | 6.2, 1.6 | 6.4, 1.6 | 6.0, 1.6 | < 0.001 |
| Urine protein * | 1.8, 1.7 | 2.2, 1.8 | 1.4, 1.5 | < 0.001 |
| Urine protein ** | 1.5 [0.2, 3] | 2.1 [0.5, 3.8] | 0.9 [0.0, 2.4] |  |

Mean, standard deviation, Value, %

* Continuous value of urine protein test by dipstick

** Semi-quantity test of urine protein test by dipstick 50% [25%, 75%],

0; -, 1; +, 2; +, 3; ++, 4; +++, 5; ++++

RD; rapid decline, SBP; systolic blood pressure, DBP; diastolic blood pressure, eGFR; estimated glomerular filtration rate, BUN; blood urea nitrogen, T-C; total cholesterol, TG; triglyceride
