# Supplementary material for: Increasing tendency of urine protein is a risk factor for rapid eGFR decline in patients with CKD: A machine learning-based prediction model by using a big database
Source: PLoS One. 2020 Sep 17;15(9):e0239262. doi: 10.1371/journal.pone.0239262 (PMC7497987; doi:10.1371/journal.pone.0239262)
Supplement: Attachment — Submitted filename: Reply to reviewers comment.docx [file pone.0239262.s003.docx]

**Reply:**

Thanks for your comments.

1. As you pointed out, we removed description from line 57 (with respect to kidney dysfunction during stages 1, 2, and 3a of CKD) and from line 283 to 288 (Further, glomerular hyperfiltration is known to lead to proteinuria and glomerular sclerosis, and subsequently result in the decline of GFR. Therefore, we attempted to reduce intra-glomerular blood pressure by prescribing medications, including renin angiotensin blockers and sodium glucose transporter-1. The strong correlation between the ESA of proteinuria and GFR decline established in the present study was proved under the assumption that the increase in urinary protein excretion reflected the exacerbation of glomerular hypertension and sclerosis.).

2. We changed all “GFR” to “eGFR”.

3. We added the description and the Reference #28.
